# Relationship between nutrient profiling and environmental impacts of Norwegian dishes

**DOI:** 10.3389/fnut.2026.1837290

**Published:** 2026-05-22

**Authors:** Gizem Aytekin-Sahin, Sigrun Henjum

**Affiliations:** 1 Department of Nutrition and Dietetics, Faculty of Health Sciences, Nuh Naci Yazgan University, Kayseri, Türkiye; 2 Department of Nursing and Health Promotion, Faculty of Health Sciences, Oslo Metropolitan University, Oslo, Norway

**Keywords:** carbon footprint, food culture, Norwegian dishes, nutrient profile, water footprint

## Abstract

**Background:**

Evaluating the nutrient profiles and environmental impacts of dishes, as well as a better understanding of their relationships, is crucial for both public health policies and individual diets. Therefore, this study aims to determine the nutrient profile and environmental impact of Norwegian dishes, and to assess the relationship between them comprehensively.

**Methods:**

A total of 163 Norwegian cuisine recipes were collected. The energy and nutrient content of the dishes, as well as their nutrient profiles calculated. In addition, their carbon footprints and water footprints were calculated based on Life Cycle Assessment (LCA) method. The Nutrient Rich Food (NRF) 9.3 index was used to calculate the nutrient profile of the dishes.

**Results:**

Main dishes contributed the most to carbon (69%) and water footprints (68%), followed by desserts (11% carbon footprint, 16% water footprint), side dishes (10% carbon footprint, 8% water footprint), and soups (10% carbon footprint, 8% water footprint). Multiple linear regression analyses showed a positive relationship between protein, vitamin B_12_, and zinc contents and the carbon footprint. Protein, fat, vitamin B_12_, and phosphorus were positively related to the water footprint. On the contrary, vitamin D content had a negative relationship with both carbon and water footprint. Correlation analysis also revealed significant correlations between all nutrients (except potassium) and the carbon and water footprints of the dishes. Moreover, the NRF 9.3 scores of the dishes were negatively correlated with both carbon and water footprint.

**Conclusion:**

The fact that main dishes have both a lower NRF 9.3 score and a higher carbon and water footprint compared to soups and side dishes, and the regression and correlation analysis results suggest that even small changes in traditional dishes without eliminating animal foods can make significant contributions to health and sustainability. However, it is important to consider nutritional and environmental impacts together in the evaluation of food systems, without ignoring the nutritional importance of animal-derived foods.

## 1 Introduction

Food systems are recognized as playing a critical role in public health and environmental sustainability, and urgent change is needed to provide healthy nutrition from sustainable food systems to a growing global population (1). In addition, the food system is currently identified as a significant contributor to environmental issues. Approximately one-third of global greenhouse gas emissions come from the food system (2). Moreover, 70% of the world’s freshwater resources are withdrawn for agricultural use (3), and over 90% of the water used in nature is allocated to food production (4). Additionally, nutrition is one of the most important factors contributing to morbidity and mortality globally (5).

Diet-related noncommunicable diseases account for more than half of all deaths globally, and data suggest that adherence to healthy dietary patterns can substantially decrease the risks of morbidity and mortality from diet-related noncommunicable diseases (5, 6). Consequently, shifting toward sustainable diets is essential to achieving the United Nations (UN) Sustainable Development Goals (SDGs) and sustaining our existence within environmental boundaries (7).

Identifying and promoting dietary patterns that enhance overall diet quality and health outcomes within planetary boundaries can be accomplished by empowering consumers to make informed choices (8). In this context, nutrient profile models play a crucial role (9). Nutrient profiling is defined as “the science of classifying or ranking foods according to their nutritional composition for preventing disease and promoting health” (10). Previous studies have shown that plant-based dietary patterns, including the Mediterranean Diet and the Nordic Diet, have a favorable nutrient profile and a lower environmental impact (11, 12). Furthermore, a previous study reported that a sustainable dietary pattern that meets dietary requirements can be achieved with lower greenhouse gas emissions, without eliminating dairy products and meats or increasing costs (13).

Recognized as a sustainable dietary pattern, the Nordic diet emphasizes a high intake of fruits and vegetables, whole grains, and fish, while limiting saturated fat, red meat, and processed meat. Canola oil is the primary source of unsaturated fat in the Nordic diet, which also includes native fruits such as berries (12). The Norwegian Food-Based Dietary Guidelines also align closely with the Nordic Diet recommendations (14). However, data from the Norwegian National Dietary Survey (NORKOST) 4 indicated that Norwegian adults consume fruit and fish at levels below the recommended levels, while their intake of red meat and processed meat exceeds the upper limit (15). Compared to the previous NORKOST study data, there has been a decrease in the consumption of bread, fruit, berries, potatoes, fish, and fish products, as well as milk, while there has been an increase in the consumption of vegetables, cheese, sugar/sweets, and unsweetened fruit juices or soft drinks (15, 16). Another study indicated that Norwegian adults have a moderate compliance rate with the Norwegian Food-Based Dietary Guidelines recommendations, scoring 65 points out of 100 (17). These findings suggest that although a healthy eating pattern has been established, individuals might struggle to adhere to it. Therefore, to encourage healthier eating habits that align with dietary guidelines, it may be beneficial to first assess the nutrient profiles and environmental impacts of different dishes. With this information, small modifications can be introduced to promote a more sustainable culinary culture.

To the best of our knowledge, no study has investigated the nutrient profiles and environmental impacts (carbon and water footprint) of Norwegian dishes. However, assessing the nutrient profiles and environmental impacts of dishes, as well as a better understanding of their relationship to each other, is crucial for both policy (e.g., food-based dietary guidelines) and consumer preferences. Therefore, this study aims to determine the relationship between the nutrient profiles and the environmental impact of various Norwegian dishes. We hypothesized that dishes with higher nutrient profile scores would be associated with lower environmental impacts. To test this hypothesis, we conducted correlation and multiple regression analyses to examine the relationships between nutritional variables and environmental indicators.

## 2 Material and methods

### 2.1 Dataset collection

Recipes were collected from governmental sources used for dietary guidance in Norway, which are considered representative of the traditional and contemporary dishes commonly consumed at the population level (18, 19). In addition, recipes were obtained from widely used Norwegian culinary websites, which reflect commonly consumed dishes and everyday cooking practices in Norway (matprat.no, gladkokken.no, and tine.no). Only recipes with clear ingredient lists and defined portion sizes were included in the analysis, resulting in a dataset of 163 dishes that reflect a variety of Norwegian cuisine across major dish categories. Norwegian dishes included in the study were given in Supplementary Table 1.

The recipes were divided into four groups: main courses, side dishes, soups, and desserts. This classification was based on the traditional roles of the dishes in Norwegian Cuisine and their classification in governmental sources. A total of 163 recipes were collected, including 47 main dishes, 71 side dishes, 13 soups, and 32 desserts. In accordance with governmental sources and traditional Norwegian cuisine, red meat, poultry, and fish dishes—typically rich in protein and serving as the primary component of a meal—were classified as *main dishes (hovedretter)*. Smaller, complementary dishes, usually containing vegetables, potatoes, or grains, were categorized as *side dishes (tilbehør)*. Liquid-based dishes, ranging from light broths to more filling soups and traditionally consumed as starters or light meals, were assigned to *the soups (supper) category*. Finally, puddings, cakes, fruit-based desserts, and milk-based sweets were grouped under *the desserts (desserter) category*.

After classifying the dishes, the composition of one serving of each dish was determined. The energy and nutrient content, nutrient profile, and carbon and water footprints of each serving were then calculated. This study did not involve human participants or animals; therefore, ethical approval was not required.

#### 2.1.1 Calculating the energy and nutrient content of Norwegian dishes

The energy and nutrient content [carbohydrates, protein, fat, saturated fatty acids (SFA), dietary fibers, vitamin B_6_, folate, vitamin B_12_, vitamin C, vitamin A, vitamin D, vitamin E, calcium, magnesium, phosphorus, potassium, sodium, iron, zinc, and added sugar] in one portion of Norwegian dishes were calculated using the Norwegian Food Composition Table (19).

#### 2.1.2 Calculating the NRF 9.3 scores of Norwegian dishes

According to World Health Organization (WHO), nutrient profiling is “the science of classifying or ranking foods according to their nutritional composition for reasons related to preventing disease and promoting health” (10). In this study, the Nutrient Rich Food (NRF) score 9.3, developed by Drewnowski (20) was used to calculate the nutrient profile of the dishes. The NRF 9.3 score is a quantitative assessment tool that aims to score the nutrient profile of a food or meal by considering the beneficial and restricted nutrients it provides per energy. The NRF approach is distinctive because it is designed as a comprehensive food guidance system rather than a rating, numbering, or labeling system. It is not restricted to individual foods, the NRF index can be used to provide nutrition education and guidance, can be applied to foods, dishes, menus, or the entire diet (20).

This score consists of three nutrients that are recommended to be consumed in limited amounts and nine nutrients that are encouraged to be consumed daily. The recommended nutrients for consumption are protein, dietary fiber, vitamin A, vitamin C, vitamin E, calcium, iron, magnesium, and potassium. In contrast, saturated fat, added sugar, and sodium should be limited. Calculations are based on a 100-kcal dish content. The formula used to calculate the NRF 9.3 score is given below:


$${\text{NRF}}_{9.3}=\int_{i=1}^{9}\left(\frac{N_{i}}{D\,V_{i}}=100\right)-\int_{j=1}^{3}\left(\frac{L_{j}}{M\,R\,V_{j}}\times 100\right)$$

N_*i*_ = amount of each positive nutrient per 100 kcal

DV_*i*_ = daily value for each qualifying nutrient

L_*j*_ = amount of each limiting (negative) nutrient per 100 kcal

MRV_*j*_ = maximum recommended value for each limiting nutrient

#### 2.1.3 Calculating the carbon and water footprint of Norwegian dishes

In this study, the environmental impact of Norwegian dishes was assessed using the Life Cycle Assessment (LCA) method, a standardized and comprehensive approach to evaluating the environmental effects of a product throughout its entire life cycle (21). In this study, a cradle-to-retail system boundary was applied, including the stages of raw material production, processing, and packaging. Stages such as transport to consumers, cooking, and post-consumption waste management were excluded due to a lack of consistent data across food items. While this approach allows standardized comparisons of foods, it may underestimate the total environmental footprint of food products across their life cycles.

Carbon footprint values were obtained from two established databases relevant to the Nordic context: the Big Climate Database, developed by the Danish Consumer Council, and the Center for International Climate Research (CICERO) Database (22, 23). Water footprint values for individual food items were obtained from the Water Footprint Network (WFN) (4, 24), which provides global average estimates of water use (in m^3^/ton) based on a comprehensive Life Cycle Assessment approach that includes blue, green, and gray water components. In this study, the total (combined) water footprint of each food item was used. These sources provide average life cycle impact factors (kg CO2 eq and m^3^/ton) for commonly consumed food items in the region, based on ISO 14040/44-compliant LCA studies.

The functional unit was defined as per portion, based on standard serving sizes provided in governmental sources. For composite dishes, the carbon and water footprint was calculated by summing the contributions of individual ingredients, each weighted by their amount in the recipe and matched with its corresponding footprint value from the databases. Ingredients that weigh less than 1% of each recipe’s total weight, such as spices, garlic, and salt, were excluded from the carbon and water footprint calculation (25). The resulting estimates reflect the upstream production impact of the dishes and enable comparison across dish categories in terms of carbon and water footprint intensity.

### 2.2 Statistical analysis

Statistical Package for Social Sciences software (Version 23.0, USA, IBM Corp., 2015) was used for statistical analysis. Data visualization was performed with GraphPad Prism software (Version 8.0, San Diego, CA, USA) and R (Version 4.4.1). Normality was assessed using the Kolmogorov–Smirnov test. Data were expressed as mean ± standard deviation (SD). Dish groups were compared by the one-way analysis of variance (ANOVA) Test. Then, according to the results of the homogeneity of variances test, Bonferroni or Tamhane’s T2 *post-hoc* tests were used. The relationship between the environmental impact of Norwegian dishes and certain variables was evaluated using Pearson’s correlation.

Multiple linear regression analysis was performed to determine the nutrients that affect the carbon footprint and water footprint of Norwegian dishes. The model used the carbon footprint and water footprint as dependent variables, while the energy and nutrient contents of the dishes were used as independent variables. The selection of variables to determine the most significant variables was done using the stepwise regression method. Stepwise regression was applied to facilitate variable selection in the presence of multiple correlated nutritional variables. In the stepwise method, variables with a significance level of *p* < 0.05 were included in the model; variables whose contribution was not statistically significant were removed. To evaluate the multicollinearity problem, VIF values were examined, and autocorrelation was checked using the Durbin–Watson test. VIF values below 5 and Durbin–Watson statistics close to 2 were considered acceptable indicators of model validity. For all statistical analyses, *p* < 0.05 values were considered statistically significant.

## 3 Results

The comparison of energy and nutrient contents of Norwegian dishes is given in Table 1. The main dishes had significantly higher energy, protein, fat, saturated fatty acids (SFA), vitamin B_12_, vitamin D, phosphorus, sodium, iron, and zinc content than the other dish groups (*p* < 0.001). In addition, the vitamin B_6_ content of main dishes (0.51 ± 0.05 mg) was significantly higher than soups (0.17 ± 0.03 mg) and desserts (0.09 ± 0.01 mg) (*p* < 0.001). In contrast, the main dishes (8.13 ± 2.15 mg) had the lowest vitamin C content compared to the other groups (*p* < 0.001). The dish group with the highest carbohydrate content was desserts (32.96 ± 10.87 g), followed by side dishes (26.18 ± 5.88 g) (*p* < 0.001). Similarly, the dish group with the highest added sugar content was also desserts (15.70 ± 8.94 g, *p* < 0.001). The dish groups with the highest dietary fiber content were side dishes (5.78 ± 3.33 g) and soups (4.57 ± 1.19 g), respectively (*p* < 0.001). It was also determined that side dishes (57.57 ± 2.67 mcg) contained significantly higher folate than main dishes (27.33 ± 3.16 mcg) and desserts (25.00 ± 3.11 mcg) (*p* < 0.001). Soups (275.41 ± 101.47 mcg) contained significantly more vitamin A than desserts (128.23 ± 21.99 mcg) (*p* = 0.001). The groups containing the highest vitamin E were desserts (2.34 ± 0.41 mg) and main dishes (2.29 ± 1.16 mg), respectively (*p* = 0.001). It was also found that main dishes (53.06 ± 18.34 mg) and side dishes (49.88 ± 12.83 mg) had the highest magnesium content compared to other dish groups (*p* < 0.001). The dish groups with the lowest potassium content compared to the other groups were desserts (319.50 ± 29.53 mg) and soups (420.15 ± 43.80 mg, *p* < 0.001). On the contrary, the calcium contents of dish groups were similar (*p* = 0.310).

**TABLE 1:** Comparison of energy and nutrient contents of Norwegian dishes (*n* = 163).

Energy and nutrients	Soups (*n* = 13)	Main dishes (*n* = 47)	Side dishes (*n* = 71)	Desserts (*n* = 32)	*p*-value*
Energy (kcal)	171.70 ± 77.28^a^	418.02 ± 100.33^b^	269.44 ± 84.36^c^	276.31 ± 132.75^c^	**< 0.001**
Carbohydrates (g)	16.27 ± 3.75^a^	10.77 ± 1.45^a^	26.18 ± 5.88^b^	32.96 ± 10.87^c^	**< 0.001**
Protein (g)	6.02 ± 1.78^a^	26.60 ± 13.51^b^	6.34 ± 3.11^a^	5.19 ± 0.63^a^	**< 0.001**
Fat (g)	8.10 ± 2.05^a^	24.92 ± 1.54^b^	14.19 ± 1.03^a^	13.11 ± 2.19^a^	**< 0.001**
SFA (g)	4.31 ± 1.31^a^	10.86 ± 5.08^b^	7.97 ± 0.59^a^	6.65 ± 1.25^a^	**< 0.001**
Dietary fiber (g)	4.57 ± 1.19^a^	1.90 ± 0.31^b^	5.78 ± 3.33^a^	2.89 ± 0.40^b^	**< 0.001**
Vitamin B_6_ (mg)	0.17 ± 0.03^a^	0.51 ± 0.05^b^	0.26 ± 0.01^ab^	0.09 ± 0.01^a^	**< 0.001**
Folate (mcg)	42.93 ± 7.92^ab^	27.33 ± 3.16^b^	57.57 ± 2.67^a^	25.00 ± 3.11^b^	**< 0.001**
Vitamin B_12_ (mcg)	0.54 ± 0.33^a^	3.86 ± 0.60^b^	0.23 ± 0.03^a^	0.45 ± 0.08^a^	**< 0.001**
Vitamin C (mg)	35.55 ± 10.43^a^	8.13 ± 2.15^b^	31.16 ± 16.68^a^	24.15 ± 7.39^a^	**< 0.001**
Vitamin A (mcg)	275.41 ± 101.47^a^	184.09 ± 16.01^ab^	269.99 ± 22.38^a^	128.23 ± 21.99^b^	**0.001**
Vitamin D (mcg)	0.63 ± 0.40^a^	2.92 ± 0.63^b^	0.82 ± 0.07^a^	0.63 ± 0.12^a^	**< 0.001**
Vitamin E (mg)	1.75 ± 0.76^ab^	2.29 ± 1.16^a^	1.26 ± 0.10^b^	2.34 ± 0.41^a^	**0.001**
Calcium (mg)	102.54 ± 31.98	83.77 ± 11.71	111.81 ± 8.55	102.33 ± 12.85	0.310
Magnesium (mg)	29.29 ± 4.29^a^	53.06 ± 18.34^b^	49.88 ± 12.83^b^	27.16 ± 3.12^a^	**< 0.001**
Phosphorus (mg)	123.02 ± 26.25^a^	393.79 ± 17.39^b^	165.81 ± 6.80^a^	127.91 ± 15.44^a^	**< 0.001**
Potassium (mg)	420.15 ± 43.80^a^	769.82 ± 36.06^b^	854.21 ± 20.21^b^	319.50 ± 29.53^a^	**< 0.001**
Sodium (mg)	290.46 ± 44.35^a^	541.45 ± 132.11^b^	477.58 ± 60.02^c^	60.13 ± 9.75^d^	**< 0.001**
Iron (mg)	0.76 ± 0.15^a^	2.27 ± 0.25^b^	1.68 ± 0.66^c^	0.80 ± 0.10^a^	**< 0.001**
Zinc (mg)	0.59 ± 0.14^a^	4.04 ± 0.51^b^	0.94 ± 0.34^a^	0.63 ± 0.46^a^	**< 0.001**
Added sugar (g)	4.69 ± 3.31^a^	0.33 ± 0.23^a^	0.62 ± 0.19^a^	15.70 ± 8.94^b^	**< 0.001**

*ANOVA test, *p* < 0.05. Parameters with different superscript letters (a, b, c, d) are statistically significantly different from each other. SFA, saturated fatty acids. Bold values indicate statistically significant differences (*p* < 0.05).

The comparison of NRF 9.3 scores and environmental impacts of Norwegian dishes is summarized in Table 2. It was determined that NRF 9.3 scores of soups (57.48 ± 15.37) and side dishes (37.03 ± 19.79) were significantly higher than main dishes (23.36 ± 17.10) and desserts (15.10 ± 5.39) (*p* < 0.001). It was also found that the mean carbon footprint (2.70 ± 2.43 kg CO_2_ eq) and water footprint (1.44 ± 1.09 m^3^/ton) of the main dishes were significantly higher than those of the other groups (*p* < 0.001).

**TABLE 2:** Comparison of nutrient profiles and environmental impacts of Norwegian dishes.

Parameters	Soups (*n* = 13)	Main dishes (*n* = 47)	Side dishes (*n* = 71)	Desserts (*n* = 32)	*p*-value*
NRF 9.3 scores	57.48 ± 15.37^a^	23.36 ± 17.10^b^	37.03 ± 19.79^a^	15.10 ± 5.39^b^	**< 0.001**
Carbon footprint (kg CO_2_ eq)	0.38 ± 0.23^a^	2.70 ± 2.43^b^	0.41 ± 0.21^a^	0.45 ± 0.25^a^	**< 0.001**
Water footprint (m^3^/ton)	0.17 ± 0.14^a^	1.44 ± 1.09^b^	0.18 ± 0.06^a^	0.34 ± 0.27^a^	**< 0.001**

*ANOVA test, *p* < 0.05. Parameters with different superscript letters (a, b) are statistically significantly different from each other. NRF, Nutrient Rich Food. Bold values indicate statistically significant differences (*p* < 0.05).

**FIGURE 1:**
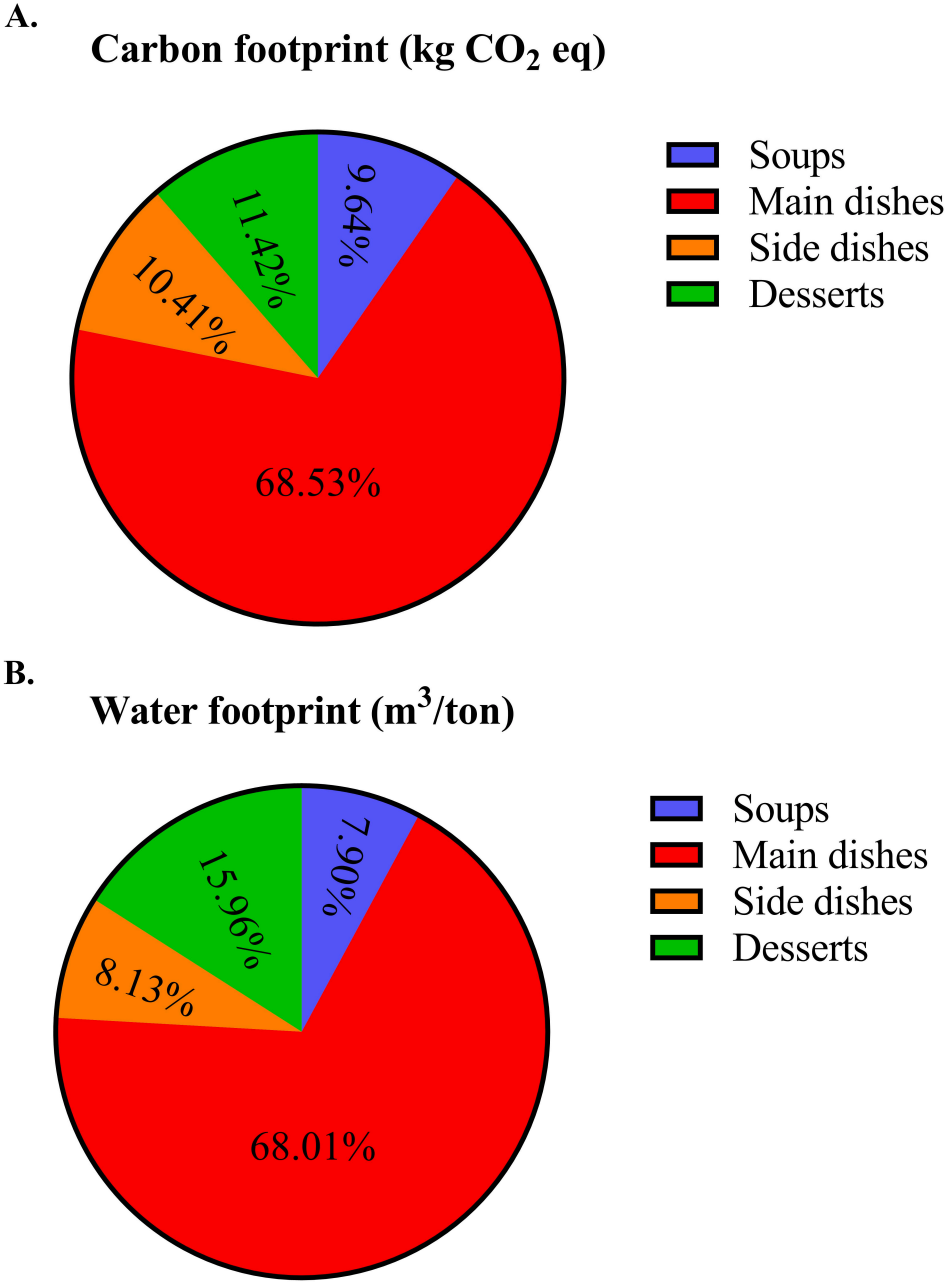
Contribution of each dish group to the total environmental impact of Norwegian dishes. Panel **(A)** shows the percentage distribution of total carbon footprint (kg CO2 eq), and panel **(B)** shows the water footprint (m^3^/ton), categorized by dish type: soups (*n* = 13), main dishes (*n* = 47), side dishes (*n* = 71), and desserts (*n* = 32). These percentages represent each dish group’s total contribution to the overall footprint across all recipes (*n* = 163), not average per-dish values.

The contribution of each dish group to the total environmental impact of Norwegian dishes is presented in Figure 1. Percentages represent each dish group’s total contribution to the overall footprint across all recipes (*n* = 163), rather than average per-dish values. The main dishes contributed the most to carbon (69%) and water footprints (68%), followed by desserts (contribution of carbon footprint: 11%, contribution of water footprint: 16%), side dishes (contribution of carbon footprint: 10%, contribution of water footprint: 8%), and soups (contribution of carbon footprint: 10%, contribution of water footprint: 8%). These contributions are influenced not only by environmental intensity but also by the number and composition of recipes within each dish category.

**FIGURE 2:**
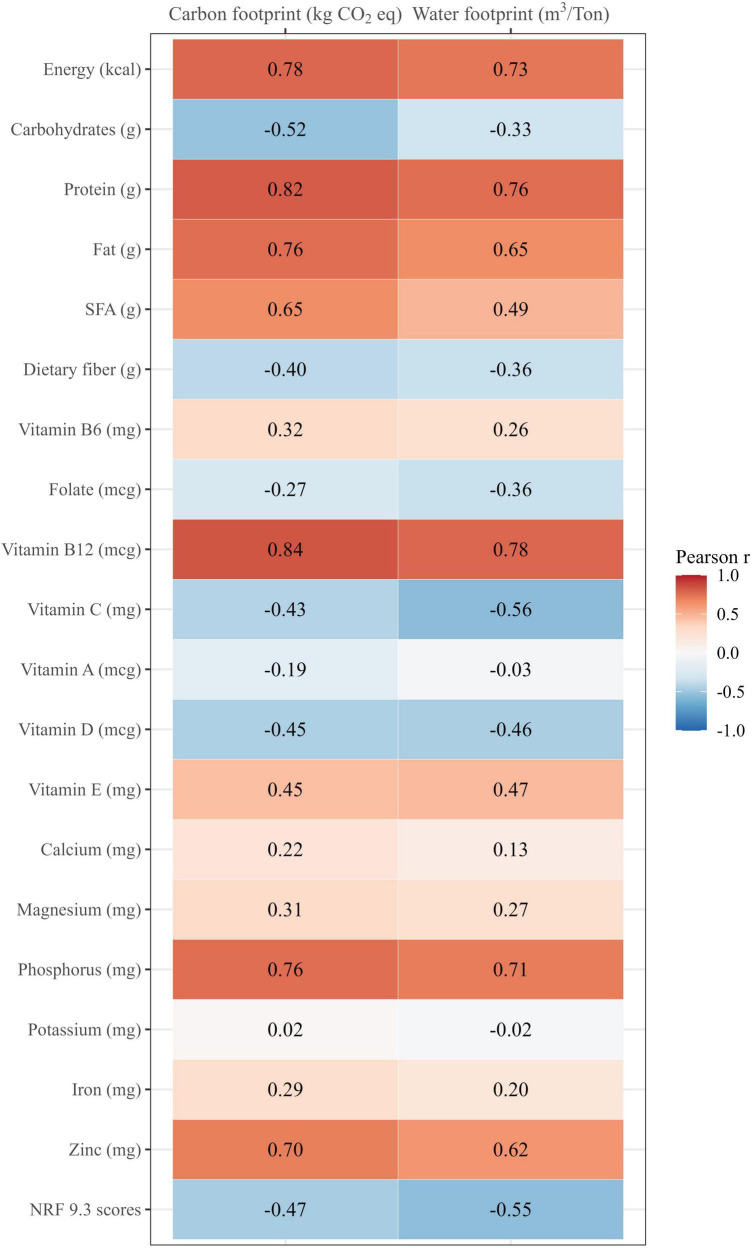
Heatmap visualization of correlation between Norwegian dishes’ environmental impact and some variables. Pearson Correlation, *p* < 0.05.

Table 3 summarizes the correlation between the environmental impact of Norwegian dishes and some variables, and Figure 2 provides a visual summary of the correlation analysis, complementing the numerical results presented in Table 3 and facilitating easier identification of key relationships. Carbon footprint showed strong positive correlations with energy (*r* = 0.78, *p* < 0.001) protein (*r* = 0.82, *p* < 0.001), fat (*r* = 0.76, *p* < 0.001), vitamin B_12_ (*r* = 0.84, *p* < 0.001), phosphorus (*r* = 0.76, *p* < 0.001), and zinc (*r* = 0.70, *p* < 0.001); moderate positive correlations with SFA (*r* = 0.65, *p* < 0.001); weak positive correlations with vitamin B_6_ (*r* = 0.32, *p* < 0.001), vitamin E (*r* = 0.45, *p* < 0.001), magnesium (*r* = 0.31, *p* < 0.001), and iron (*r* = 0.29, *p* < 0.001); and very weak positive correlation between calcium (*r* = 0.22, *p* = 0.006). In contrast, there was a moderate negative correlation between carbon footprint and carbohydrates (*r* = −0.52, *p* < 0.001), weak negative correlations with dietary fiber (*r* = −0.40, *p* < 0.001), folate (*r* = −0.27, *p* = 0.001), vitamin C (*r* = −0.43, *p* < 0.001), vitamin D (*r* = −0.45, *p* < 0.001), and NRF 9.3 scores (*r* = −0.47, *p* < 0.001), and very weak negative correlation with vitamin A (*r* = −0.19, *p* = 0.016).

**TABLE 3:** Correlation between Norwegian dishes environmental impact and some variables.

Parameters*	Carbon footprint (kg CO_2_ eq)	Water footprint (m^3^/ton)
Energy (kcal)	***r* = 0.78**	***r* = 0.73**
	***p* < 0.001**	***p* < 0.001**
Carbohydrates (g)	***r* = −0.52**	***r* = −0.33**
	***p* < 0.001**	***p* < 0.001**
Protein (g)	***r* = 0.82**	***r* = 0.76**
	***p* < 0.001**	***p* < 0.001**
Fat (g)	***r* = 0.76**	***r* = 0.65**
	***p* < 0.001**	***p* < 0.001**
SFA (g)	***r* = 0.65**	***r* = 0.49**
	***p* < 0.001**	***p* < 0.001**
Dietary fiber (g)	***r* = −0.40**	***r* = −0.36**
	***p* < 0.001**	***p* < 0.001**
Vitamin B_6_ (mg)	***r* = 0.32**	***r* = 0.26**
	***p* < 0.001**	***p* < 0.001**
Folate (mcg)	***r* = −0.27**	***r* = −0.36**
	***p* = 0.001**	***p* < 0.001**
Vitamin B_12_ (mcg)	***r* = 0.84**	***r* = 0.78**
	***p* < 0.001**	***p* < 0.001**
Vitamin C (mg)	***r* = −0.43**	***r* = −0.56**
	***p* < 0.001**	***p* < 0.001**
Vitamin A (mcg)	***r* = −0.19**	*r* = −0.03
	***p* = 0.016**	*p* = 0.739
Vitamin D (mcg)	***r* = −0.45**	***r* = −0.46**
	***p* < 0.001**	***p* < 0.001**
Vitamin E (mg)	***r* = 0.45**	***r* = 0.47**
	***p* < 0.001**	***p* < 0.001**
Calcium (mg)	***r* = 0.22**	*r* = 0.132
	***p* = 0.006**	*p* = 0.093
Magnesium (mg)	***r* = 0.31**	***r* = 0.27**
	***p* < 0.001**	***p* < 0.001**
Phosphorus (mg)	***r* = 0.76**	***r* = 0.71**
	***p* < 0.001**	***p* < 0.001**
Potassium (mg)	*r* = 0.02	*r* = −0.02
	*p* = 0.830	*p* = 0.772
Iron (mg)	***r* = 0.29**	***r* = 0.20**
	***p* < 0.001**	***p* = 0.010**
Zinc (mg)	***r* = 0.70**	***r* = 0.62**
	***p* < 0.001**	***p* < 0.001**
NRF 9.3 scores	***r* = −0.47**	***r* = −0.55**
	***p* < 0.001**	***p* < 0.001**

*Pearson correlation, *p* < 0.05. NRF, Nutrient Rich Food; SFA, saturated fatty acids. Bold values indicate statistically significant differences (*p* < 0.05).

Water footprint had strong positive correlations with energy (*r* = 0.73, *p* < 0.001) protein (*r* = 0.76, *p* < 0.001), vitamin B_12_ (*r* = 0.78, *p* < 0.001), and phosphorus (*r* = 0.71, *p* < 0.001); moderate positive correlations with fat (*r* = 0.65, *p* < 0.001) and zinc (*r* = 0.62, *p* < 0.001); weak positive correlations with SFA (*r* = 0.49, *p* < 0.001), vitamin B_6_ (*r* = 0.26, *p* < 0.001), vitamin E (*r* = 0.47, *p* < 0.001), and magnesium (*r* = 0.27, *p* < 0.001); and very weak positive correlations with iron (*r* = 0.20, *p* = 0.010). On the contrary, there was a moderate negative correlation between water footprint and vitamin C (*r* = −0.56, *p* < 0.001) and NRF 9.3 scores (*r* = −0.55, *p* < 0.001), a weak negative correlation with carbohydrates (*r* = −0.33, *p* < 0.001), dietary fiber (*r* = −0.36, *p* < 0.001), folate (*r* = −0.36, *p* < 0.001), and vitamin D (*r* = −0.46, *p* < 0.001) (Table 3 and Figure 2).

Multiple linear regression models for carbon footprint prediction are given in Table 4. The model was found to be significant [Adjusted *R*^2^ = 0.593, *F*_(4,158)_ = 60.043, *p* < 0.001]. Notably, the model revealed a suppressor effect for vitamin D; although vitamin D was not significantly associated with carbon footprint in univariate analysis, it showed a significant inverse association after controlling for other variables. According to this model, a one-gram increase in protein was associated with a 0.029-unit increase in the carbon footprint (95% CI = 0.009; 0.048, *p* = 0.004). Similarly, a one-mcg increase in vitamin B_12_ was associated with a 0.49-unit increase in the carbon footprint (95% CI = 0.338; 0.642, *p* < 0.001), and a one-mg increase in zinc was associated with a 0.145-unit increase in the carbon footprint (95% CI = 0.029; 0.261, *p* = 0.015). In contrast, a 1-mcg increase in vitamin D was associated with a 0.424-unit reduction in carbon footprint (95% CI = −0.540; −0.279, *p* < 0.001). Univariate linear regression analysis revealed that protein, vitamin B_12_, and zinc contents were significantly and positively associated with carbon footprint, whereas vitamin D content was not significantly associated with carbon footprint (Supplementary Table 2).

**TABLE 4:** Multiple linear regression models for carbon footprint prediction.

Model	Carbon footprint (kg CO_2_ eq)				
	B	95% CI for B	β	*T*	*p*-value*
Protein (g)	0.029	0.009; 0.048	0.274	2.924	**0.004**
Vitamin B_12_ (mcg)	0.490	0.338; 0.642	0.805	6.366	**< 0.001**
Vitamin D (mcg)	−0.424	−0.540; −0.279	−0.661	−5.750	**< 0.001**
Zinc (mg)	0.145	0.029; 0.261	0.207	2.471	**0.015**
	Adjusted R^2^ = 0.593, ***p* < 0.001**				

*Multiple linear regression analysis, *p* < 0.05. Dependent variable: carbon footprint, independent variables: protein, vitamin B_12_, vitamin D, zinc. All VIF values were below 2, indicating no significant multicollinearity. The Durbin–Watson statistics were 2.284, suggesting independence of residuals. B, unstandardized regression coefficient; CI, confidence interval; β, standardized coefficient. Bold values indicate statistically significant differences (*p* < 0.05).

Table 5 shows multiple linear regression models for water footprint prediction. The model was found to be significant [Adjusted R^2^ = 0.606, *F*_(5,157)_ = 50.810, *p* < 0.001]. According to this model, a one-gram increase in protein was associated with a 0.029-unit rise in the water footprint (95% CI = 0.017; 0.042, *p* < 0.001), and a one-gram increase in fat was associated with a 0.017-unit rise in the water footprint (95% CI = 0.007; 0.027, *p* = 0.001). Similarly, a one-mcg increase in vitamin B_12_ was associated with a 0.283-unit rise in the water footprint (95% CI = 0.208; 0.358, *p* < 0.001), and a one-mg increase in phosphorus was associated with a 0.001-unit rise in water footprint (95% CI = 0.0002; 0.003, *p* = 0.028). In contrast, a 1-mcg increase in vitamin D was associated with a 0.261-unit reduction in water footprint (95% CI = −0.331; −0.191, *p* < 0.001).

**TABLE 5:** Multiple linear regression models for water footprint prediction.

Model	Water footprint (m^3^/ton)				
	B	95% CI for B	β	*T*	*p*-value*
Protein (g)	0.029	0.017; 0.042	0.568	4.571	**< 0.001**
Fat (g)	0.017	0.007; 0.027	0.237	3.477	**0.001**
Vitamin B_12_ (mcg)	0.283	0.208; 0.358	0.950	7.476	**< 0.001**
Vitamin D (mcg)	−0.261	−0.331; −0.191	−0.830	−7.410	**< 0.001**
Phosphorus (mg)	0.001	0.0002; 0.003	0.301	2.218	**0.028**
	Adjusted R^2^ = 0.606, ***p* < 0.001**				

*Multiple linear regression analysis, *p* < 0.05. Dependent variable: water footprint, independent variables: protein, fat, vitamin B_12_, vitamin D, phosphorus. All VIF values were below 2, indicating no significant multicollinearity. The Durbin–Watson statistics were 2.474, suggesting independence of residuals. B, unstandardized regression coefficient; CI, confidence interval; β, standardized coefficient. Bold values indicate statistically significant differences (*p* < 0.05).

To clarify the individual effects of each nutrient on carbon and water footprint, results of univariate linear regression analyses performed are also presented in Supplementary Tables 2, 3.

## 4 Discussion

As far as we know, this is the first study to evaluate the relationship between NRF 9.3 scores and environmental impact of Norwegian dishes. The main findings of the study revealed significant relationships between the NRF 9.3 scores, energy, almost all nutrients, and environmental impact parameters. Current findings suggest that including more plant-based dishes, such as soups and side dishes that are rich in fiber and micronutrients and have a lower environmental footprint, into traditional dish patterns, while simultaneously reducing the frequency or portion size of animal-based main dishes may be an important step toward achieving both healthier and more sustainable diets.

Although the traditional Nordic diet is considered a healthy and sustainable dietary pattern, it is acknowledged that the Western/sweet dietary pattern is prevalent in Scandinavian countries, including Norway (25, 26). However, there is significant evidence that diets rich in vegetables, fruits, whole grains, nuts, legumes, low-fat dairy products, and seafood—while low in red and processed meats, refined grains, and sugar-sweetened foods and beverages—can significantly reduce the risk of all-cause mortality, cardiovascular disease, obesity, type 2 diabetes, as well as several cancer types (26). Therefore, it is also crucial to understand the dietary pattern and culinary culture.

Nutrient profiles enable comparative and balanced evaluation of dishes (20). In the current study, the NRF 9.3 score was used to assess the nutrient profiles of the dishes. It was found that the NRF 9.3 scores of soups and side dishes was higher than that of main dishes and desserts. Soups and vegetable-containing side dishes are generally thought to have lower energy density and higher micronutrient content. Additionally, previous studies, although not focusing directly on dishes, have demonstrated that vegetables and legumes have a higher nutrient profile score due to their higher NRF 9.3-associated micronutrient (such as vitamin A, vitamin C, vitamin E, calcium, iron, magnesium, and potassium) and fiber content, and have lower fat and sugar content (27, 28). On the other hand, main dishes and, especially, desserts are generally expected to have lower NRF 9.3 scores, because they contain higher amounts of fat, sugar, and energy.

Currently, humanity encounters the challenge of providing sustainable food for the global population, which is expected to reach approximately ten billion by 2050 (29). To manage this challenge, it is essential to calculate the environmental impact of culinary cultures, as computational approaches based on recipe data can provide an opportunity to create sustainable recipes while minimizing environmental impact (30). The present study’s results showed that the average carbon and water footprint of main dishes was significantly higher than those of other dish groups. However, it is important to remember that the analysis only covers the stages from cradle-to-retail, and the results reflect only the impacts at the production level. The energy used for cooking and consumer-level waste can alter total environmental impacts and vary significantly between dishes. It has also been observed that the dish group making the highest contribution to the carbon and water footprint is the main dish, and red meat dishes account for 51.1% of the main dishes included in the dataset. Lamb dishes, which are considered relatively more sustainable in Norway (31), made up only 4.3% of the main dishes. Previous studies have reported that the production of animal products requires more energy, water, and land use, and results in higher greenhouse gas emissions compared to plant products (32, 33). In contrast, side dishes, soups, and desserts contain more plant-based ingredients, such as vegetables, legumes, and grains, resulting in lower environmental impacts for these categories (34, 35). Animal-sourced foods, when consumed in moderation, can positively impact health by providing high-quality protein and essential micronutrients such as vitamin B_12_, iron, and zinc, many of which are limited or less bioavailable in plant-based diets (36). There is also a prejudice among consumers that plant-based foods are not tasty enough (37). Considering all these reasons, more sustainable cuisines might be created with recipes rich in plant foods without eliminating animal foods from the diet.

To help contextualize environmental impact estimates, sustainable dietary patterns can be considered. For example, adherence to the EAT-Lancet Commission’s planetary health diet has been reported to be associated with a carbon footprint of approximately 2.56 kg CO2-eq and a water footprint of approximately 0.31 m^3^ (38). In contrast, studies on typical Norwegian diets estimate the carbon footprint to be 3.8–4.8 kg CO2-eq (38, 39), and the average water footprint to be 0.58 m^3^ (38) both higher than the EAT-Lancet diet. These metrics show that the carbon footprints of individual dishes, particularly main dishes (2.70 ± 2.43 kg CO_2_-eq) containing red meat in our study, are significant relative to the environmental goals of the overall diet.

To identify independent associations between nutrient composition and environmental indicators, multivariable regression analyses were conducted. In these analyses, protein, vitamin B_12_, and zinc, which are generally found in higher amounts in animal-derived foods, were positively associated with the carbon footprint of dishes, while, similarly, protein, fat, vitamin B_12_, and phosphorus content were positively associated with the water footprint of dishes. These findings are consistent with the literature, which reports that animal-based nutrients are generally associated with greater environmental impact (34, 35, 40, 41) In univariate analyses (Supplementary Tables 2, 3), most nutrients also showed significant positive associations with carbon and water footprints; however, these results reflect bivariate relationships that may be confounded by intercorrelations among nutrients. Notably, some differences were observed between univariate and multivariable findings—for example, vitamin D was not significantly associated with the carbon footprint in univariate analysis—highlighting the importance of interpreting independent nutrient–environment relationships primarily on the basis of multivariable regression models, which provide a more robust assessment of each nutrient’s independent contribution to environmental impact.

In addition, according to the results of the energy and nutrient content analysis, the dish group that contains the lowest amount of nutrients, which are negatively correlated with the environmental impact parameters, is the main dish. These findings showed that plant-based ingredients, particularly vegetables, fruits, grains, and legumes, are both rich in nutrients and have a low environmental impact (40, 42). Plant-based foods, particularly vegetables and fruits, are rich in vitamin C and folate (43), and have a low greenhouse gas emission and water use profile (41, 44). Similarly, foods rich in dietary fiber, such as grains, legumes, and vegetables, are generally more environmentally sustainable than animal products (45). Furthermore, when vitamin A content is primarily derived from plant-based carotenoids (e.g., carrots, squash, spinach), the carbon footprint of these foods is low, which supports this negative correlation. This indicates that plant-based dishes with high micronutrient content should be preferred more not only from a nutritional perspective but also from an environmental perspective. However, the negative relationship between vitamin D and carbon and water footprint seems unexpected at first glance. Among animal foods containing vitamin D, oily fish (e.g., salmon, herring) and eggs stand out. Such foods generally have a lower environmental footprint than other animal products, such as red meat or dairy products (5, 41). Consequently, the negative relationship between vitamin D and environmental footprint may be since this vitamin is obtained from relatively environmentally friendly animal sources. According to the current dataset, fish dishes accounted for 42.5% of the main dishes. Among the rich in vitamin D dishes were fish dishes, such as vegetable-breaded pollock, crispy baked salmon, and boiled salmon. The recipes did not include any fortified dairy products or supplements. Since vitamin D content in the dishes primarily comes from fish, this inverse association may reflect the relatively lower environmental footprint of these sources compared to red meat. While the current study found a negative association, further research is needed to assess the specific contribution of fortified dairy products or other high-impact sources to vitamin D levels in these dishes. In addition, although the environmental footprint values used in the current study do not differentiate between wild-caught and farmed fish, previous research indicates that these production systems can have differing environmental impacts depending on species, feed input, and catch methods (46). Therefore, this limitation should be considered when interpreting the observed association between vitamin D content and environmental indicators. In univariate regression analysis, most nutrients from the multiple regression analysis also showed significant positive associations with carbon and water footprints. However, vitamin D was found to have no significant association with carbon footprint but showed a positive association with water footprint. This suggests that the association of certain nutrients with environmental indicators may be confounded when analyzed in isolation. Therefore, multivariate regression results provide a more robust understanding of the independent impact of each nutrient on environmental impact.

Correlation analyses revealed a significant negative association between the NRF 9.3 scores of dishes and their carbon and water footprints, suggesting that dishes with higher nutrient profile scores tend to be associated with lower environmental impacts. As these findings are based on bivariate associations, they should be interpreted as indicative rather than causal relationships. Similar results are also available in the literature. Masset et al. (47) stated that diets with low energy density and high nutrient density are more environmentally sustainable. Another study conducted in Türkiye found that the NRF 9.3 scores of hospital menus was negatively correlated with their carbon and water footprints (48). These findings suggest that within the Norwegian culinary context, sustainable dietary models that address a better nutrient profile and low environmental impact goals should be developed. Our study provides significant evidence on this issue, particularly in the context of Norwegian food culture. At this point, vegan or vegetarian diets may provide greater environmental benefits but are unlikely to be adopted in practice and may reduce the intake and/or bioavailability of some essential nutrients (e.g., iron, zinc and vitamin B_12_) (49). Therefore, in the context of Norwegian food culture, it would be more realistic to aim for adequate, balanced consumption of animal-based foods while increasing consumption of plant-based foods (such as the Nordic diet).

This study has several limitations. First, environmental impact data are based on average values, and variations in production methods, geographical origin, and seasonality are not fully reflected. In addition to the stated limitations, these factors also represent sources of uncertainty that should be considered when interpreting the results. Moreover, since the system’s boundary is limited to the cradle-to-retail, the results should be interpreted within this context. Excluding the cooking and food waste stages may lead to underestimating or misrepresenting total environmental impacts, especially for recipes that require energy-intensive preparation or generate significant waste. Nevertheless, because the same methodological scope was applied to all recipes, relative comparisons between dishes remain valid. Future studies may benefit from including post-retail stages to provide a more comprehensive assessment. From a conceptual perspective, some limitations should also be acknowledged. Due to the nature of the study, recipe-based ingredient lists were used; actual consumption quantities, portion sizes, or consumer behavior were not considered. A strength of the study is the use of a recipe dataset derived from governmental sources that reflects commonly consumed Norwegian dishes at the population level. At the same time, although these sources provide a standardized and policy-relevant representation of national dietary patterns, the recipes may not fully capture regional, seasonal, or individual-level variation in current Norwegian consumption practices. Another strength of the study includes the comprehensive approach to assessing both the nutrient profile and environmental impacts, such as carbon and water footprints, on a large Norwegian dish sample. The combined use of correlation and multiple regression analyses increased the reliability of the findings regarding the relationship between nutrition and environmental sustainability. A strength of the study is the use of a recipe dataset derived from governmental sources that reflects commonly consumed Norwegian dishes at the population level. At the same time, although these sources provide a standardized and policy-relevant representation of national dietary patterns, the recipes may not fully capture regional, seasonal, or individual-level variation in current Norwegian consumption practices. Another strength of the study includes the comprehensive approach to assessing both the nutrient profile and environmental impacts, such as carbon and water footprints, on a large Norwegian dish sample. The combined use of correlation and multiple regression analyses increased the reliability of the findings regarding the relationship between nutrition and environmental sustainability.

## 5 Conclusion

Main findings have shown that the main dishes contributed the most to carbon and water footprints compared to other dish groups. In addition, multiple linear regression analyses showed a positive relationship between protein, vitamin B_12_, and zinc contents and the carbon footprint. Protein, fat, vitamin B_12_, and phosphorus were positively related to the water footprint. Vitamin D content had a negative relationship with both carbon and water footprint. Correlation analysis also revealed significant correlations between all nutrients (except potassium) and the carbon and water footprints of the dishes. Moreover, the NRF 9.3 scores of the dishes were negatively correlated with both carbon and water footprint.

These results highlight the importance of integrating both nutrient profile and environmental sustainability parameters in food system evaluations without ignoring the moderate consumption of animal-based foods in the Norwegian food system. The findings contribute to the growing body of evidence supporting the promotion of nutrient-dense, plant-based foods to mitigate environmental impacts. In practice, including traditional dishes with higher nutrient density and a lower environmental footprint, such as vegetable-based soups or fish-based main courses, in dietary guidelines could support both health and sustainability. Furthermore, encouraging the inclusion of such dishes in institutional meal plans, such as those in schools, hospitals, or nursing homes, could inform public procurement policies aiming to align nutrition and environmental priorities. However, such implications should be interpreted with caution, as Life Cycle Assessment coefficients, recipe composition, and food systems may differ substantially across countries, and the present findings are specific to the Norwegian food system. These implications should also be considered in light of the cradle-to-retail system boundary applied in this study, as dishes requiring energy-intensive cooking or generating higher levels of post-retail food waste may have different overall environmental impacts.

Future research should incorporate additional environmental indicators, such as land use and biodiversity loss, and account for actual consumption patterns to enhance the robustness and applicability of sustainable dietary frameworks. While this study focused specifically on Norwegian dishes, future research may benefit from comparing the nutritional and environmental characteristics of dishes across different culinary traditions to provide broader insights into sustainable diet patterns. In addition, this study focused on dish-level analysis rather than whole dietary patterns; therefore, comparisons with internationally accepted diets were beyond its scope. Future research may benefit from integrating such comparisons to provide a broader perspective on sustainable dietary patterns. Moreover, based on these findings, it is recommended that public health strategies be implemented to promote nutrient-dense and environmentally sustainable food choices, support plant-based dishes, and incorporate environmental impact considerations into national dietary guidelines and public education programs.

## Data Availability

The datasets presented in this article are not readily available as the project is still ongoing and the dataset will be used for future studies. Requests to access the datasets should be directed to Gizem Aytekin-Sahin, gasahin@nny.edu.tr.
