# Supplementary material for: Relationship between nutrient profiling and environmental impacts of Norwegian dishes
Source: Front Nutr. 2026 May 22;13:1837290. doi: 10.3389/fnut.2026.1837290 (PMC13237826; doi:10.3389/fnut.2026.1837290)
Supplement: Supplementary file 1 [file Table_1.pdf]

Supplementary Table S1. Norwegian dishes included in the study.

No.	Dish name in Norwegian	Dish name in English	Dish type	Ingredients
1	Nypesuppe	Rosehip soup	soup	rosehip, water, sugar, potato starch
2	Spinatsuppe	Spinach soup	soup	spinach, milk, butter, flour, salt, pepper
3	Fiskesuppe	Fish soup	soup	cod, potato, carrot, leek, cream, fish stock
4	Ertesuppe	Pea soup	soup	dried peas, onion, carrot, celery, herbs
5	Betasuppe	Beetroot soup	soup	beetroot, onion, vinegar, sugar, vegetable stock, cream
6	Sjampinjongsuppe	Mushroom soup	soup	mushroom, onion, butter, cream, garlic, vegetable stock
7	Fruktsuppe	Fruit soup	soup	mixed fruits (e.g., apples, berries), water, sugar, cinnamon
8	Blomkål- og brokkolisuppe	Cauliflower and broccoli soup	soup	cauliflower, broccoli, onion, garlic, cream, vegetable stock
9	Saftsuppe med byggryn	Juice soup with barley groats	soup	berry juice, barley groats, sugar
10	Blåbærsuppe	Blueberry soup	soup	blueberry, water, sugar, potato starch
11	Klar grønnsaksuppe	Vegetable soup	soup	mixed vegetables (carrot, potato, cabbage, leek), vegetable stock, herbs
12	Kremede suppe	Creamy soup	soup	cream, butter, flour, broth
13	Jordbærsuppe	Strawberry soup	soup	strawberry, water, sugar, lemon juice, starch
14	Husets kjøttdeiggryte	Meatball stew	main dishes	ground beef, onion, carrot, potato, tomato paste, beef stock, cream, salt, pepper
15	Røkt svinekam med soppsaus	Smoked pork loin with mushroom sauce	main dishes	smoked pork loin, mushroom, cream, butter, onion, flour, meat stock

16	Kokt laks med rømmesaus	Boiled salmon with sour cream sauce	main dishes	salmon, water, salt, sour cream, dill, lemon juice
17	Lammestek med rosmarinsaus	Roast lamb with rosemary sauce	main dishes	lamb, rosemary, garlic, butter, potatoes, lamb stock
18	Kjøttpudding med brun saus	Meat pudding with brown sauce	main dishes	ground beef, milk, egg, onion, flour, nutmeg, brown sauce (stock, butter, flour)
19	Bakt torsk med urtesmør	Baked cod with herb butter	main dishes	cod, butter, parsley, dill, garlic, lemon, salt
20	Lys Lapskaus med lam	Norwegian stew with lamb	main dishes	lamb, potato, carrot, leek, cabbage, pepper, broth
21	Stekt flesk med duppe	Roasted pork with creamy sauce	main dishes	pork, cream, butter, onion, meat stock, salt, pepper
22	Oksesteik med timiansaus	Steak beef with thyme sauce	main dishes	beef steak, thyme, butter, cream, garlic, beef stock
23	Kalkunsteaklet med bearnaise	Turkey steak with béarnaise sauce	main dishes	turkey breast, butter, egg yolk, tarragon, vinegar, shallot, lemon juice
24	Sprøbakt laks med smør	Crispy baked salmon with butter	main dishes	salmon, butter, salt, pepper, lemon, dill
25	Røkte pølser med brun saus	Smoked sausages with brown sauce	main dishes	smoked sausages, butter, flour, meat stock, onion, brown sauce
26	Fiskepudding med hvit saus	Fish pudding with white sauce	main dishes	cod, milk, egg, potato starch, butter, flour, milk, nutmeg
27	Kalvefrikadeller med fløtesaus	Veal meatballs with cream sauce	main dishes	ground veal, onion, egg, milk, breadcrumbs, cream, butter, meat stock
28	Kylling med kremet urtesaus	Chicken with creamy herb sauce	main dishes	chicken, cream, butter, garlic, parsley, thyme, chicken stock
29	Pinnekjøtt	Dried and salted lamb ribs	main dishes	salted and dried lamb ribs, water (for steaming)
30	Sosekjøtt	Meat with sauce	main dishes	beef, onion, carrot, bay leaf, pepper, brown sauce
31	Fiskekaker med brun saus	Fish cakes with brown sauce	main dishes	cod, potato starch, milk, onion, butter, brown sauce
32	Kjøttkaker med brun saus	Meatballs with brown sauce	main dishes	ground beef, onion, egg, milk, breadcrumbs, butter, brown sauce

33	Kokte laks med rømmesaus	Boiled salmon with sour cream sauce	main dishes	salmon, water, salt, sour cream, dill, lemon juice
34	Viltkarbonader med soppsaus	Venison chops with mushroom sauce	main dishes	venison, mushroom, cream, butter, onion, meat stock
35	Pai med salat	Pie with salad	main dishes	pie crust (flour, butter), filling meat, egg, cream, mixed salad (lettuce, tomato, cucumber)
36	Ferskt kjøtt med sursøt saus	Fresh meat with sweet and sour sauce	main dishes	beef, cabbage, carrot, potato, vinegar, sugar, meat stock
37	Seibiff med løksjy	Pollock steak with onion sauce	main dishes	pollock, onion, butter, flour, fish stock, salt, pepper
38	Kålruletter med hvit saus	Cabbage rolls with white sauce	main dishes	cabbage leaves, ground beef, rice, onion, milk, butter, flour, milk
39	Kylling i karri	Chicken in curry	main dishes	chicken, curry powder, cream, onion, garlic, chicken stock
40	Skinkesteik med brun saus	Ham steak with brown sauce	main dishes	ham, butter, flour, meat stock, onion, brown sauce
41	Pølser	Norwegian-style sausage	main dishes	sausages
42	Fiskepudding med smør	Fish pudding with butter	main dishes	cod, milk, egg, potato starch, butter
43	Karbonader med peppersaus	Norwegian meat patties with pepper sauce	main dishes	ground beef, onion, egg, breadcrumbs, butter, cream, pepper sauce
44	Fiskegrateng med smør	Fish gratin with butter	main dishes	white fish, macaroni, milk, butter, flour, egg, breadcrumbs
45	Fiskeboller med hvit saus	Fish balls with white sauce	main dishes	white fish, milk, potato starch, eggs, butter, flour, milk
46	Lettpanert torsk med sitron og pepper	Breaded cod with lemon and pepper	main dishes	cod, breadcrumbs, egg, flour, butter or oil, lemon, black pepper
47	Karbonader med fløtesaus	Norwegian meat patties with cream sauce	main dishes	ground beef, onion, egg, breadcrumbs, cream, butter, meat stock
48	Kyllinggryte med sjampinjong og løk	Chicken stew with mushrooms and onions	main dishes	chicken, mushroom, onion, cream, butter, garlic, chicken stock

49	Svinenakke med brun saus	Pork neck with brown sauce	main dishes	pork neck, butter, flour, meat stock, onion, brown sauce
50	Lammeboller med brunsaus	Lamb meatballs with brown sauce	main dishes	ground lamb, onion, egg, breadcrumbs, milk, butter, brown sauce
51	Grønnsakspanert sei	Vegetable breaded pollack	main dishes	pollock, breadcrumbs, egg, flour, mixed vegetables (carrot, peas), butter
52	Lysing med hollandaisesaus	Lysing with hollandaise sauce	main dishes	lysing, egg yolk, butter, lemon juice, salt
53	Biff Lindstrøm med soppsaus	Steak Lindstrøm with mushroom sauce	main dishes	ground beef, onion, pickled beetroot, caper, egg, mushroom, cream, butter
54	Sprøbakt fiskerullade med spinat	Crispy baked fish roulade with spinach	main dishes	white fish, spinach, cream cheese or cream, egg, breadcrumbs, butter
55	Nakkekoteletter med brun saus	Neck chops with brown sauce	main dishes	pork neck chops, butter, flour, meat stock, onion, brown sauce
56	Fersk skrei med sandefjorsmør	Fresh skrei with sandefjord butter	main dishes	skrei, butter, cream, lemon juice, parsley
57	Skogsgryte med sjampinjong og løk	Forest stew with mushroom and onion	main dishes	Beef, mushroom, onion, cream, butter, thyme, meat stock
58	Ovnsbakt laks med rømmedressing	Oven baked salmon with sour cream dressing	main dishes	salmon, sour cream, dill, lemon juice, salt, pepper
59	Lapskaus mørk	Norwegian meat stew	main dishes	beef, potato, carrot, onion, cabbage, bay leaf, pepper, meat stock
60	Medisterkaker	Norwegian pork meatballs	main dishes	ground pork, onion, milk, breadcrumbs, egg, salt, pepper, nutmeg
61	Ovnsbakte rotgrønnsaker	Oven-roasted root vegetables	side dishes	potato, carrot, parsnip, rutabaga, onion, oil, salt, pepper
62	Rotmos og poteter	Mashed root vegetables and potatoes	side dishes	potato, carrot, rutabaga, butter, milk, salt
63	Potetmos og herregårdsblanding	Mashed potatoes and country-style mixed vegetables	side dishes	potato, carrot, pea, corn, butter, milk, salt

64	Potetmoso g brokkoliblanding	Mashed potatoes and broccoli mix	side dishes	potato, broccoli, butter, milk, salt
65	Potetmos og amerikansk blanding	Mashed potatoes and American vegetable mix	side dishes	potato, carrot, pea, corn, green bean, butter, milk, salt
66	Potetmos og kokte gulrøtter	Mashed potatoes and boiled carrots	side dishes	potato, carrot, butter, milk, salt
67	Potetmos og rotgrønnsaker	Mashed potatoes and root vegetables	side dishes	potato, carrot, rutabaga, parsnip, butter, milk, salt
68	Potetmos og surkål	Mashed potatoes and sauerkraut	side dishes	potato, sauerkraut (fermented cabbage), butter, milk, salt
69	Potetmos og kålrotstappe	Mashed potatoes and roasted turnips	side dishes	potato, turnip, butter, oil, salt
70	Potetmos og aspargesbønner	Mashed potatoes and asparagus beans	side dishes	potato, green bean, butter, milk, salt
71	Potetmos og bønnestuing	Mashed potatoes and bean stew	side dishes	potato, bean, onion, butter, milk, salt
72	Potetmos og kålstuing	Mashed potatoes and cabbage stew	side dishes	potato, cabbage, butter, milk, salt
73	Potetmos og ertestuing	Mashed potatoes and pea stew	side dishes	potato, pea, butter, milk, salt
74	Potetmos og bukettblanding	Mashed potatoes and mixed vegetable bouquet	side dishes	potato, carrot, cauliflower, broccoli, butter, milk, salt
75	Potetmos og rødkål	Mashed potatoes and pickled red cabbage	side dishes	potato, red cabbage, vinegar, sugar, butter, milk, salt
76	Potetmos og dampet gulrot	Mashed potatoes and steamed carrots	side dishes	potato, carrot, butter, milk, salt
77	Potetmos og norsk blanding	Mashed potatoes and Norwegian vegetable mix	side dishes	potato, carrot, pea, cabbage, butter, milk, salt
78	Potetmos og selleripuré	Mashed potatoes and celery puree	side dishes	potato, celery root, butter, milk, salt
79	Potetmos, brokkoli og blomkål	Mashed potatoes, broccoli and cauliflower	side dishes	potato, broccoli, cauliflower, butter, milk, salt
80	Potetmos og blomkålblanding	Mashed potatoes and cauliflower mix	side dishes	potato, cauliflower, butter, milk, salt
81	Kokte poteter og brokkoliblanding	Boiled potatoes and broccoli mix	side dishes	potato, broccoli, butter, salt
82	Kokte potete og amerikansk blanding	Boiled potatoes and American vegetable mix	side dishes	potato, carrot, peas, corn, green bean, butter, salt

83	Kokte poteter og kokte gulrøtter	Boiled potatoes and boiled carrots	side dishes	potato, carrot, butter, salt
84	Kokte poteter og rotgrønnsaker	Boiled potatoes and root vegetables	side dishes	potato, carrot, rutabaga, parsnip, butter, salt
85	Kokte poteter og grønnsakstuing	Boiled potatoes and vegetable stew	side dishes	potato, carrot, cabbage, peas, onion, butter, salt
86	Kokte poteter og surkål	Boiled potatoes and sauerkraut	side dishes	potato, sauerkraut (fermented cabbage), butter, salt
87	Kokte poteter og kålrotstappe	Boiled potatoes and roasted turnips	side dishes	potato, turnip, butter, oil, salt
88	Kokte poteter og aspargesbønner	Boiled potatoes and asparagus beans	side dishes	potato, green bean, butter, salt
89	Kokte poteter og bønnestuing	Boiled potatoes and bean stew	side dishes	potato, bean, onion, butter, salt
90	Kokte poteter og kålstuing	Boiled potatoes and cabbage stew	side dishes	potato, cabbage, butter, salt
91	Kokte poteter og ertestuing	Boiled potatoes and pea stew	side dishes	potato, peas, butter, salt
92	Kokte poteter og bukettblanding	Boiled potatoes and mixed vegetable bouquet	side dishes	potato, carrot, broccoli, cauliflower, butter, salt
93	Kokte poteter og rødkål	Boiled potatoes and pickled red cabbage	side dishes	potato, red cabbage, vinegar, sugar, butter, salt
94	Kokte poteter og norsk blanding	Boiled potatoes and Norwegian vegetable mix	side dishes	potato, carrot, peas, cabbage, butter, salt
95	Kokte poteter og selleripuré	Boiled potatoes and celery puree	side dishes	potato, celery root, butter, milk, salt
96	Kokte poteter, brokkoli og blomkål	Boiled potatoes, broccoli and cauliflower	side dishes	potato, broccoli, cauliflower, butter, salt
97	Kokte poteter og blomkålblanding	Boiled potatoes and cauliflower mix	side dishes	potato, cauliflower, butter, salt
98	Fløtepoteter og brokkoliblanding	Creamy potatoes and broccoli mix	side dishes	potato, broccoli, cream, butter, salt
99	Fløtepoteter og amerikansk blanding	Creamy potatoes and American vegetable mix	side dishes	potato, cream, butter, carrot, peas, corn, green bean, salt
100	Fløtepoteter og kokte gulrøtter	Creamy potatoes and boiled carrots	side dishes	potato, cream, butter, carrot, salt
101	Fløtepoteter og rotgrønnsaker	Creamy potatoes and root vegetables	side dishes	potato, cream, butter, carrot, rutabaga, parsnip, salt

102	Fløtepoteter og grønnsakstuing	Creamy potatoes and vegetable stew	side dishes	potato, cream, butter, carrot, cabbage, peas, onion, salt
103	Fløtepoteter og surkål	Creamy potatoes and sauerkraut	side dishes	potato, cream, butter, sauerkraut (fermented cabbage), salt
104	Fløtepoteter og kålrotstappe	Creamy potatoes and roasted turnips	side dishes	potato, cream, butter, turnip, salt
105	Fløtepoteter og aspargesbønner	Creamy potatoes and asparagus beans	side dishes	potato, cream, butter, green bean, salt
106	Fløtepoteter og bønnestuing	Creamy potatoes and bean stew	side dishes	potato, cream, butter, bean, onion, salt
107	Fløtepoteter og kålstuing	Creamy potatoes and cabbage stew	side dishes	potato, cream, butter, cabbage, salt
108	Fløtepoteter og ertestuing	Creamy potatoes and pea stew	side dishes	potato, cream, butter, peas, salt
109	Fløtepoteter og bukettblanding	Creamy potatoes and mixed vegetable bouquet	side dishes	potato, cream, butter, carrot, broccoli, cauliflower, salt
110	Fløtepoteter og rødkål	Creamy potatoes and pickled red cabbage	side dishes	potato, cream, butter, red cabbage, vinegar, sugar, salt
111	Fløtepoteter og norsk blanding	Creamy potatoes and Norwegian vegetable mix	side dishes	potatoes, cream, butter, carrot, peas, cabbage, salt
112	Fløtepoteter og selleripuré	Creamy potatoes and celery puree	side dishes	potatoes, cream, butter, celery root, salt
113	Fløtepoteter, brokkoli og blomkål	Creamy potatoes, broccoli and cauliflower	side dishes	potato, cream, butter, broccoli, cauliflower, salt
114	Fløtepoteter og blomkålblanding	Creamy potatoes and cauliflower mix	side dishes	potato, cream, butter, cauliflower, salt
115	Røstipoteter og brokkoliblanding	Roasted potatoes and broccoli mix	side dishes	potato, broccoli, butter, salt, pepper
116	Røstipoteter og amerikansk blanding	Roasted potatoes and American vegetable mix	side dishes	potato, carrot, peas, corn, green bean, butter, salt
117	Røstipoteter og kokte gulrøtter	Roasted potatoes and boiled carrots	side dishes	potato, carrot, butter, salt
118	Røstipoteter og rotgrønnsaker	Roasted potatoes and root vegetables	side dishes	potato, carrot, parsnip, rutabaga, butter, salt, pepper
119	Røstipoteter og grønnsakstuing	Roasted potatoes and vegetable stew	side dishes	potato, carrot, cabbage, peas, onion, butter, salt

120	Røstipoteter og surkål	Roasted potatoes and sauerkraut	side dishes	potato, sauerkraut (fermented cabbage), butter, salt
121	Røstipoteter og kålrotstappe	Roasted potatoes and roasted turnips	side dishes	potato, turnip, butter, salt
122	Røstipoteter og aspargesbønner	Roasted potatoes and asparagus beans	side dishes	potato, green bean, butter, salt
123	Røstipoteter og bønnestuing	Roasted potatoes and bean stew	side dishes	potato, bean, onion, butter, salt
124	Røstipoteter og kålstuing	Roasted potatoes and cabbage stew	side dishes	potato, cabbage, onion, butter, salt
125	Røstipoteter og ertestuing	Roasted potatoes and pea stew	side dishes	potato, peas, onion, butter, salt
126	Røstipoteter og bukettblanding	Roasted potatoes and mixed vegetable bouquet	side dishes	potato, carrot, broccoli, cauliflower, butter, salt
127	Røstipoteter og rødkål	Roasted potatoes and pickled red cabbage	side dishes	potato, red cabbage, vinegar, sugar, butter, salt
128	Røstipoteter og norsk blanding	Roasted potatoes and Norwegian vegetable mix	side dishes	potato, carrot, peas, cabbage, butter, salt
129	Røstipoteter og selleripuré	Roasted potatoes and celery puree	side dishes	potato, celery root, butter, salt
130	Røstipoteter, brokkoli og blomkål	Roasted potatoes, broccoli and cauliflower	side dishes	potato, broccoli, cauliflower, butter, salt, pepper
131	Røstipoteter og blomkålblanding	Roasted potatoes and cauliflower mix	side dishes	potato, cauliflower, butter, salt
132	Sjokoladekake	Chocolate cake	dessert	flour, sugar, cocoa powder, egg, butter, milk, baking powder
133	Gulrotkake	Carrot cake	dessert	carrot, flour, sugar, egg, oil, cinnamon, baking powder
134	Eplegrøt med dessertfløte	Apple porridge with whipped cream	dessert	apple, sugar, water, cinnamon, potato starch, whipped cream
135	Bringebærgele med vaniljesaus	Raspberry jelly with custard sauce	dessert	raspberry, sugar, gelatin, water, vanilla custard (milk, egg, sugar)
136	Tropisk Fromasj	Tropical mousse	dessert	fruit juice, gelatin, sugar, cream
137	Blåbærkompott med vaniljesaus	Blueberry compote with custard sauce	dessert	blueberry, sugar, water, potato starch, vanilla custard

138	Ostekake	Cheesecake	dessert	cream cheese, sugar, egg, cream, biscuit base (crushed biscuits, butter)
139	Rødgrøt med dessertfløte	Red porridge with whipped cream	dessert	red berries, sugar, water, potato starch, whipped cream
140	Sjokoladepudding med vaniljesaus	Chocolate pudding with custard sauce	dessert	milk, cocoa, sugar, cornstarch, vanilla custard
141	Plommekompott	Plum compote	dessert	plum, sugar, water
142	Langpannekake med blåbær	Blueberry pancake	dessert	flour, milk, egg, sugar, blueberry, butter
143	Sjokoladefromasj	Chocolate mousse	dessert	chocolate, egg, sugar, cream, butter
144	Appelsingele med vaniljesaus	Orange jelly with custard sauce	dessert	orange juice, sugar, gelatin, vanilla custard
145	Skogsbærgrøt med dessertfløte	Wildberry porridge with whipped cream	dessert	mixed berries (blueberries, raspberries, strawberries), sugar, water, potato starch, whipped cream
146	Cappuccinofromasj	Cappuccino mousse	dessert	coffee, sugar, gelatin, cream, milk
147	Vaniljepudding med bringebærsaus	Vanilla pudding with raspberry sauce	dessert	milk, sugar, vanilla, cornstarch, raspberry
148	Aprikoskompott	Apricot compote	dessert	apricot, sugar, water
149	Smuldrepai med epler og is	Crumb pie with apples and ice cream	dessert	apple, flour, butter, sugar, cinnamon, ice cream
150	Kirsebærfromasj	Cherry mousse	dessert	cherry, sugar, gelatin, cream
151	Husets fruktsalat	Fruit salad	dessert	mixed fruits (apple, banana, orange, grapes), sugar, citrus juice
152	Semulepudding med rød saus	Semolina pudding with red sauce	dessert	semolina, milk, sugar, red berry sauce
153	Sviskekompott	Prune compote	dessert	prunes, sugar, water
154	Solbærgrøt	Blackcurrant porridge	dessert	blackcurrants, sugar, water, potato starch
155	Mandelpudding med rød saus	Almond pudding with red sauce	dessert	almond, milk, sugar, gelatin, red berry sauce

156	Jordbærkompott	Strawberry compote	dessert	strawberry, sugar, water
157	Krokanfromasj	Crocan mousse	dessert	caramelized sugar (crocan), cream, gelatin, egg
158	Gelé med vaniljesaus	Jelly with custard	dessert	fruit jelly (juice, sugar, gelatin), vanilla custard
159	Solbærkompott	Blackcurrant compote	dessert	blackcurrants, sugar, water
160	Bakte epler med vaniljesaus	Baked apples with custard	dessert	apples, sugar, cinnamon, butter, vanilla custard
161	Kirsebærkompott	Cherry compote	dessert	cherry, sugar, water
162	Karamellpudding	Caramel pudding	dessert	milk, egg, sugar, caramelized sugar
163	Bakte bjørnebær med vaniljesaus	Baked blackberries with custard	dessert	blackberries, sugar, butter, vanilla custard
