# Supplementary material for: Relationship between nutrient profiling and environmental impacts of Norwegian dishes
Source: Front Nutr. 2026 May 22;13:1837290. doi: 10.3389/fnut.2026.1837290 (PMC13237826; doi:10.3389/fnut.2026.1837290)
Supplement: Supplementary file 2 [file Table_2.docx]

**Supplementary Table S2.** Univariate linear regression results for carbon footprint prediction.

| **Predictor** | **B** | **95% CI for B** | **β** | ***t*** | ***p*-value^*^** | **Adjusted R^2^** |
| --- | --- | --- | --- | --- | --- | --- |
| Protein (g) | 0.070 | 0.058; 0.082 | 0.669 | 11.413 | **< 0.001** | 0.444 |
| Vitamin B₁₂ (mcg) | 0.287 | 0.203; 0.370 | 0.471 | 6.766 | **< 0.001** | 0.217 |
| Vitamin D (mcg) | 0.090 | -0.009; 0.189 | 0.141 | 1.802 | 0.073 | 0.014 |
| Zinc (mg) | 0.453 | 0.370; 0.536 | 0.646 | 10.751 | **< 0.001** | 0.414 |

^*^Linear regression analysis, p < 0.05**. Dependent variable:** Carbon footprint, **independent variables**: Protein, Vitamin B_12_, Vitamin D, Zinc. **Abbreviations:** B, unstandardized regression coefficient; CI, Confidence Interval; β, standardized coefficient.
