# Supplementary material for: Relationship between nutrient profiling and environmental impacts of Norwegian dishes
Source: Front Nutr. 2026 May 22;13:1837290. doi: 10.3389/fnut.2026.1837290 (PMC13237826; doi:10.3389/fnut.2026.1837290)
Supplement: Supplementary file 3 [file Table_3.docx]

**Supplementary Table S3.** Univariate linear regression results for water footprint prediction.

| **Predictor** | **B** | **95% CI for B** | **β** | ***t*** | ***p*-value^*^** | **Adjusted R^2^** |
| --- | --- | --- | --- | --- | --- | --- |
| Protein (g) | 0.035 | 0.029; 0.041 | 0.684 | 11.895 | **< 0.001** | 0.464 |
| Fat (g) | 0.028 | 0.018; 0.038 | 0.387 | 5.318 | **< 0.001** | 0.144 |
| Vitamin B_12_ (mcg) | 0.150 | 0.110; 0.190 | 0.504 | 7.402 | **< 0.001** | 0.249 |
| Vitamin D (mcg) | 0.060 | 0.012; 0.108 | 0.190 | 2.453 | **< 0.015** | 0.030 |
| Phosphorus (mg) | 0.004 | 0.003; 0.004 | 0.609 | 9.736 | **< 0.001** | 0.367 |

^*^Linear regression analysis, p < 0.05**. Dependent variable:** Carbon footprint, **independent variables**: Protein, Fat, Vitamin B_12_, Vitamin D, Phosphorus. **Abbreviations:** B, unstandardized regression coefficient; CI, Confidence Interval; β, standardized coefficient.
